# Ionic Liquid-Modified Copper for the Enhanced Thermal Conductivity and Mechanical Properties of Epoxy Resin/Expanded Graphite Composites

**DOI:** 10.1021/acsomega.4c06340

**Published:** 2024-09-23

**Authors:** Yan-Chun Li, Na Chu, Fan-Long Jin, Soo-Jin Park

**Affiliations:** † Department of Chemistry and Pharmaceutical Engineering, Jilin Institute of Chemical Technology, Jilin City 132022, People’s Republic of China; ‡ Department of Chemistry, Inha University, Michuhol-gu, Incheon 22212, South Korea; § Department of Polymer Materials, Jilin Institute of Chemical Technology, Jilin City 132022, People’s Republic of China

## Abstract

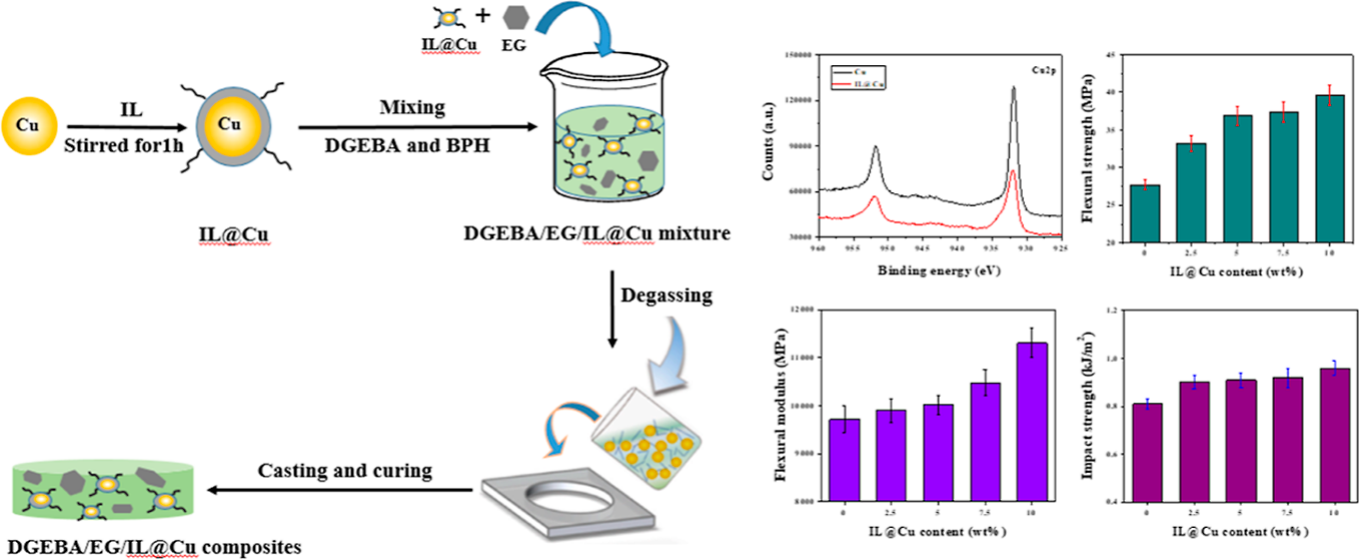

In this study, diglycidylether of bisphenol A (DGEBA)/expanded graphite (EG)/copper (Cu) powder composites with high thermal conductivity were prepared for use as thermal interface materials. To construct an excellent thermally conductive network, the Cu surface was modified using the ionic liquid 1-ethyl-3-methyl imidazolium dicyanamide. In addition, the effect of the Cu content on the thermal conductivity, thermal stability, flexural properties, impact strength, and morphologies of the DGEBA/EG/Cu composites was investigated. The results indicated that the addition of 10 wt % Cu increased the thermal conductivity of the composites from 7.35 to 9.86 W/(m·K). Conversely, the thermal stability of the composites decreased with the addition of Cu. The flexural strength and impact strength of the composites increased from 27.9 MPa and 0.81 kJ/m^2^ to 39.6 MPa and 0.96 kJ/m^2^, respectively, as the Cu content increased from 0 to 10 wt %. Moreover, the flexural modulus of the composites increased from 9632 to 11,309 MPa with the addition of 10 wt % Cu. Scanning electron microscopy analysis of the DGEBA/EG/Cu composites revealed sheet-shaped blocks with numerous microcracks on the fracture surfaces.

## 1 Introduction

Recently, with the rapid advancement of microelectronic techniques, electronic devices have been swiftly developed toward miniaturization, high integration, and multifunctionality. During the operation of the integrated circuit, the heat generated must be dissipated efficiently to ensure that the electronic devices function safely. Therefore, electronic packaging materials should have good heat dissipation efficiency to efficiently transfer the heat of the integrated circuit as quickly as possible.^1−6^

Epoxy-based composites have competitive advantages such as high mechanical properties, excellent adhesion, outstanding corrosion resistance, good dimensional and thermal stabilities, and excellent electrical insulation properties, which enable their wide use for the packaging of electronic devices. However, epoxy resin exhibits poor thermal conductivity due to its low crystallinity, which limits its wider applications in the microelectronic packaging field.^7−10^ To improve the thermal conductivity of epoxy resin, adding one or several kinds of highly thermally conductive materials is the most effective and simple method to form a thermally conductive pathway in an epoxy matrix. Such thermally conductive composites are generally prepared by using carbon materials, metals, and ceramics.^11−16^

Among such carbon materials, expanded graphite (EG) is a loose, porous, modified graphite that is prepared from natural flake graphite. EG has a layered structure similar to that of natural flake graphite, with layered planes of sp^2^-hybridized carbon atoms, each bonded with three other carbon atoms. Therefore, EG exhibits very high thermal and electrical conductivities. In addition, EG has large interlayer spacing and contains a tremendous variety of different pore sizes.^17−23^

However, to achieve high thermal conductivity with the addition of a single thermally conductive filler in polymer-based composites, a large amount of filler is usually required. This requirement not only makes the preparation process difficult but also causes filler agglomeration and bubble formation in the polymer matrix, thereby greatly reducing the mechanical properties of the resulting composites. In addition, combining two fillers to prepare polymer-based composites not only capitalizes on the advantages of the individual fillers but can also promote a synergistic effect between the two fillers.^24,25^

Among metal nanoparticles that have been used as thermally conductive fillers, copper (Cu) nanoparticles have a high thermal conductivity similar to that of silver but are much less expensive. However, Cu nanoparticles exhibit high chemical activity and react easily with oxygen in the air to form a copper oxide layer on the surface. Moreover, Cu nanoparticles tend to agglomerate in a polymer matrix and have poor dispersion in the polymer matrix because of their large specific surface area.^26−28^ Therefore, the surfaces of Cu nanoparticles are modified by using various surface modification methods including both chemical and physical methods to prevent the oxidation and aggregation of these nanoparticles. Treating Cu nanoparticles with organic compounds such as coupling agents can achieve moderate stability and improve the compatibility of the nanoparticles with the polymer matrix, but this approach diminishes the conductive properties of the resulting composites.^29−31^

An ionic liquid (IL) is a molten salt consisting of a large organic cation and a smaller organic or inorganic anion. ILs are widely used in the fields of electrochemistry, organic synthesis, chemical separation, and materials preparation due to their unique characteristics such as low volatility, nonflammability, high ionic conductivity, good thermal and electrical conductivity, and high thermal stability.^32−34^

To date, several researchers have developed highly thermally conductive epoxy-based composites using various copper powders and surface-modified copper powders.^35−40^ Ahn et al. investigated the thermal conductivity of epoxy composites filled with TiO_2_-coated copper nanowires.^35^ Their results showed that the epoxy/TiO_2_-coated copper nanowire composites exhibited thermal conductivities ranging from 0.2 to 1.1248 W/(m·K). Doan et al. enhanced the thermal conductivity of epoxy composites by introducing cellulose fibers, which have a relatively aligned structure, with copper flakes embedded in the fiber surface layer.^36^ The thermal conductivity of the resulting composite reached 1.4 W/m·K at 36.4 wt % filler content. Yim et al. studied the effect of hybrid fillers such as silver-plated EG, graphite, and copper powder on the thermal conductivity of epoxy composites.^37^ Their results indicated that the thermal conductivity of the obtained composites was enhanced to 1.71 W/m·K by the addition of a 10 phr hybrid filler. Lopez-Barajas et al. improved the thermal conductivity of epoxy resins up to 1.95 W/m·K with the addition of 15 wt % hybrid filler.^38^ In their study, a hybrid filler composed of graphene and Cu nanoparticles was obtained by high-energy mechanical wet milling with the addition of ethylene glycol. X-ray photoelectron spectroscopy (XPS) findings suggested that a certain amount of Cu was bonded to the graphene surface and formed copper–carbon bonds such as Cu=C and Cu–C. Li et al. prepared a hybrid filler through the deposition copper nanoparticles on the surface of a graphene aerogel and used it to improve the thermal conductivity of epoxy resin.^39^ The thermal conductivity of the as-prepared composite with 2.5 wt % hybrid filler was 2.09 W/m·K, which is approximately 11 times that of pristine epoxy resin. Wang et al. applied a coating of nickel nanoparticles on the surface of Cu nanowires as a thermally conductive filler to prepare epoxy-based composites.^40^ Their results indicated that the thermal conductivity of the composites along the direction of the magnetic field was 2.90 W/m·K at 9 vol % nickel-coated copper nanowires, which is 12.81 times that of pristine epoxy resin.

In this study, we develop a facile strategy to fabricate diglycidylether of bisphenol A (DGEBA)-based composites with high thermal conductivity using EG and Cu powder as thermally conductive hybrid fillers via hot blending and compression-curing processes. The Cu surface was modified using IL 1-ethyl-3-methyl imidazolium dicyanamide. The imidazolium group in this IL is more favorable for heat conduction than quaternary ammonium-based and phosphorus-based ILs due to its multielectron resonance. Cationic imidazolium and anionic dicyanamide are commonly used as hardeners for the curing of epoxy resin. Therefore, both functional groups have the potential to cross-link with epoxy resin, which contributes to the formation of a stable interface between the Cu powder and DGEBA matrix. These factors improve the dispersion of Cu particles in the DGEBA matrix, thereby increasing the thermal conductivity and mechanical properties of the DGEBA/EG/Cu composites. In addition, the effect of the Cu content on the thermal conductivity, thermal properties, flexural properties, impact strength, and morphology of the DGEBA/EG/Cu composites was investigated.

## 2 Experimental Section

### 2.1 Materials

**Figure 1:**
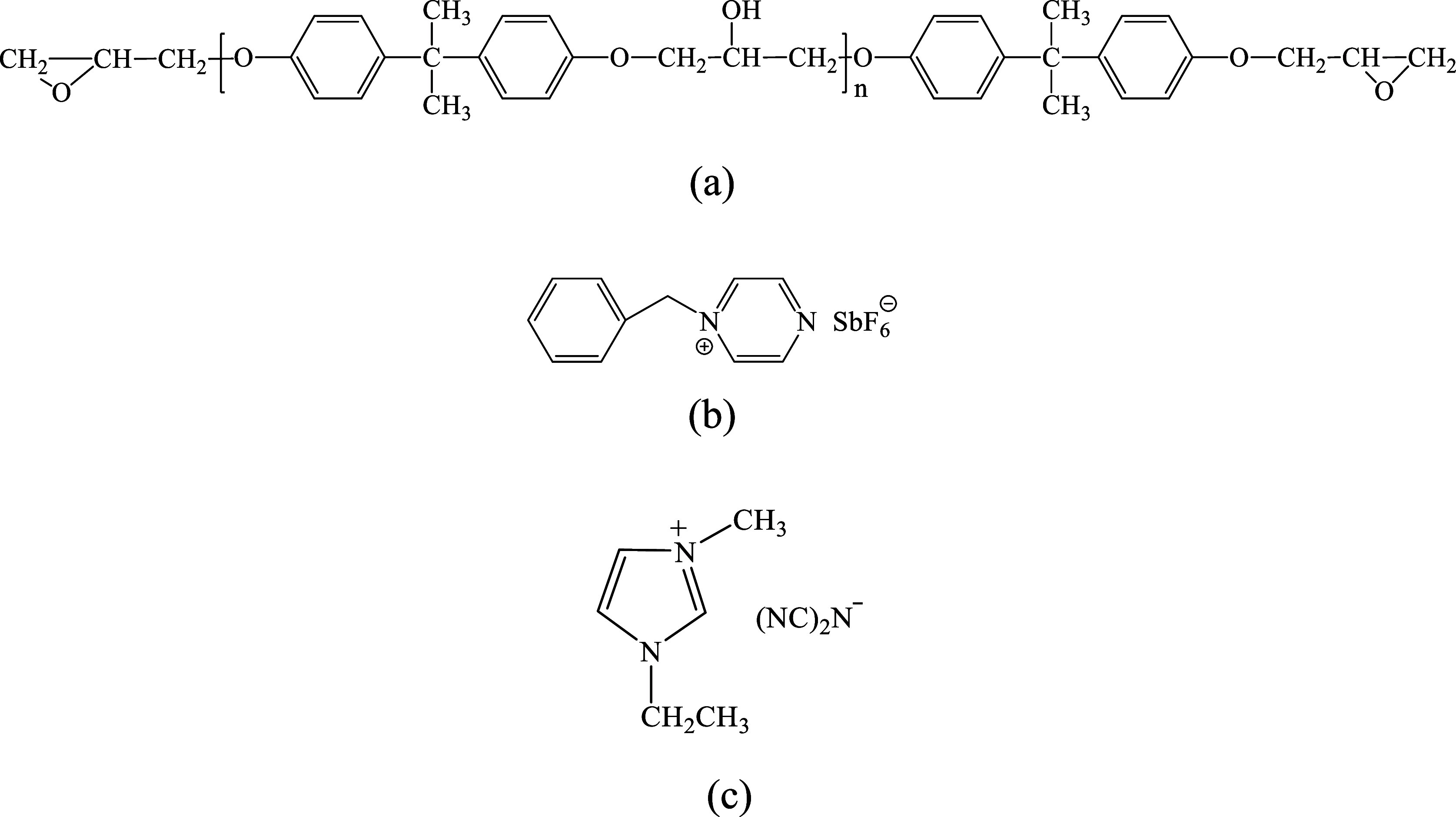
Chemical structures of (a) DGEBA, (b) BPH, and (c) the IL.

DGEBA with an epoxy equivalent weight of 184–195 g/mol was supplied by Nantong Xingchen Synthetic Material Co., Ltd. *N*-benzylpyrazinium hexafluoroantimonate (BPH), used as a thermally latent initiator of epoxy resin, was synthesized according to a previous report.^24^ EG with a particle size of 40 mesh, carbon content of 98–99%, expansion rate of 100–400, and thermal conductivity of 300 W/m·K was obtained from Jiangxi Shuobang New Material Technology Co., Ltd. Copper particles with a size of 50 nm were supplied by Zhongxin New Materials. The IL 1-ethyl-3-methyl imidazolium dicyanamide, which was used for surface modification of the copper particles, was obtained from the Lanzhou Institute of Chemical Physics. The chemical structures of DGEBA, BPH, and the IL are shown in Figure 1. Anhydrous ethanol was supplied by Sinopharm Chemical Reagent Co., Ltd. All chemicals were of analytical grade and were used without further purification.

### 2.2 Surface Modification of the Cu Powder

The IL (20 g) was dispersed in anhydrous alcohol (100 mL) and mixed evenly to prepare an IL-alcohol solution. Cu powder (30 g) was added to the IL solution, and the mixture was mixed evenly with stirring and then ultrasonically sonicated for 1 h. After the ultrasound treatment, the mixture was filtered and then dried at 35 °C for 3 h in a vacuum oven to obtain the IL-modified Cu. For convenience, IL-modified Cu is denoted as IL@Cu in this study.

### 2.3 Preparation of DGEBA/EG and DGEBA/EG/IL@Cu Composites

The desired amounts of DGEBA and BPH were mixed using a stirrer at 50 °C for 30 min, ultrasonicated for 10 min, and dried in a vacuum oven. Then, the desired amounts of EG and IL@Cu were added to the DGEBA/BPH system and mixed in a mixer at 80 °C for 30 min. The mixture was injected into a preheated mold sprayed with a mold release agent. The mold was compression-cured at temperatures of 120, 150, and 200 °C under a pressure of 5 MPa for 1 h. The specimens were cut to suitable dimensions for subsequent thermal and mechanical tests.

### 2.4 Characterization and Measurements

Fourier transform infrared (FTIR) spectra of Cu, IL, and IL@Cu were recorded using a spectrometer (Tensor II, BRUKER Company, Germany) and KBr pellets. The surface properties of Cu and IL@Cu were evaluated using XPS (Thermo ESCALAB 250) with a monochromatic Al Kα source and a passing energy of 20 eV. The surface morphologies of Cu and IL@Cu were investigated via high-resolution scanning electron microscopy (HR-SEM; Hitachi, SU 8010). Energy-dispersive X-ray spectroscopy (EDX) and SEM were performed to evaluate the presence of an IL on the EG surface.

The thermal conductivities of DGBA/EG/IL@Cu composites were measured using a thermal conductivity tester (WNK-100) following the GB/T 10294-2008 standard. For this test, the sample size of each sample was 5 × 10 × 30 mm^3^. The thermal conductivity values were obtained by taking the average of three experimental values.

The thermal stabilities of the composites were evaluated via thermogravimetric analysis (TGA; TA Instruments, Q50) from 30 to 800 °C at a heating rate of 10 °C/min under a nitrogen atmosphere.

The flexural properties of the composites were investigated via a three-point bending test using a mechanical testing apparatus (WDW 3010) according to the GB/T 9341-2008 standard. The sample size for this test was 4 × 10 × 80 mm^3^, and the cross-head speed was maintained at 2 mm/min. The flexural strength (σ_*f*_) and elastic modulus (*E*_b_) values were calculated using the following equations
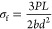
1
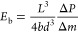
2where *P* is the applied load (in N), *L* is the span length (in mm), *b* is the specimen width (in mm), *d* is the specimen thickness (in mm), Δ*P* is the change in force in the linear portion of the load–deflection curve (in N), and Δ*m* is the corresponding change in deflection (in mm). The flexural strength and modulus values were obtained by taking the average of five experimental values.

The impact strengths of the composites were measured using an Izod impact tester (TP04G-AS1) according to the GB/T 1843-2008 standard. The sample size for this test was 4 × 10 × 50 mm^3^. The impact strength values were obtained by taking the average of five experimental values.

The morphologies of the composites after the impact strength tests were examined by using SEM (JEOL, JSM-7610FPLUS, Hitachi, S4800).

## 3 Results and Discussion

### 3.1 Characterization of the IL@Cu

**Figure 2:**
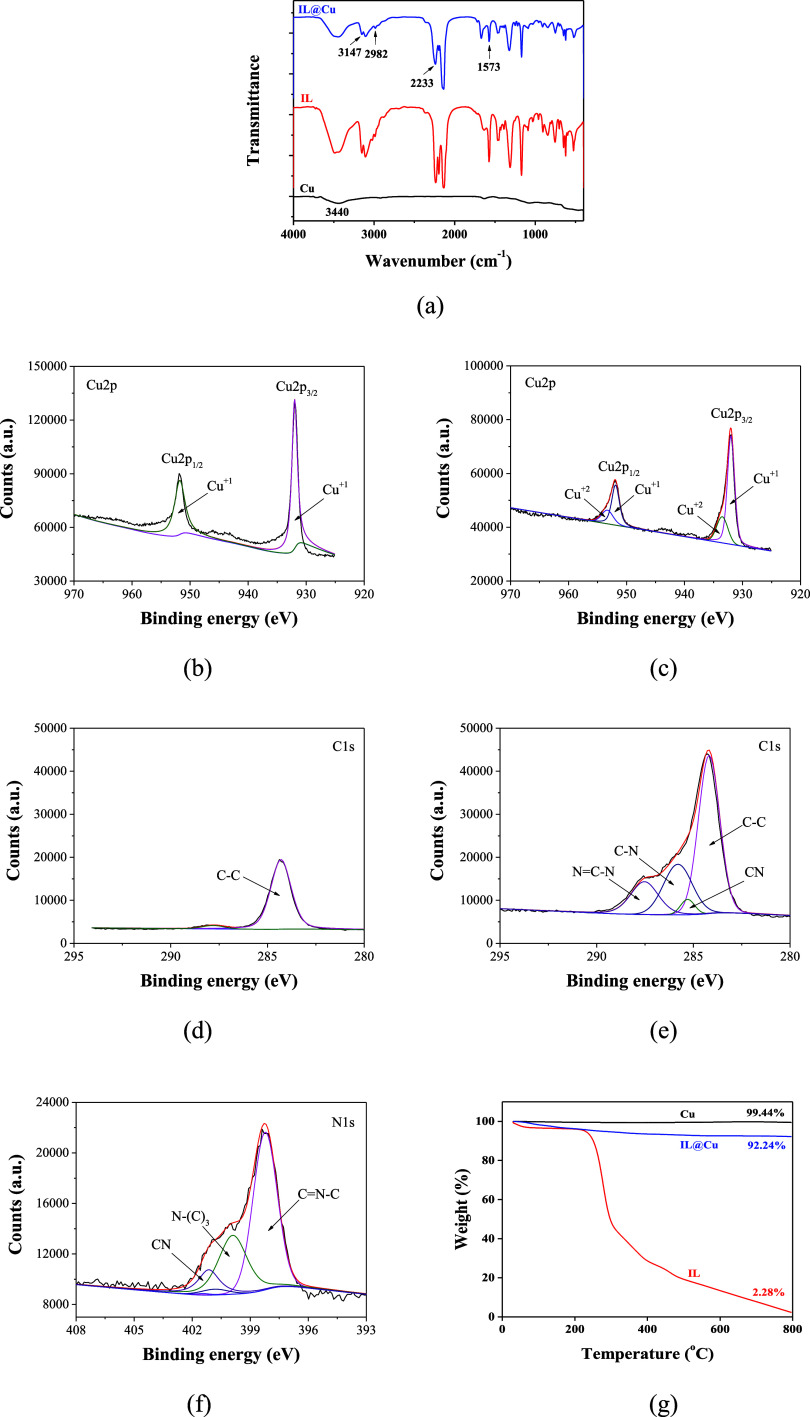
(a) FTIR spectra of Cu and IL@Cu. (b–f) High-resolution XPS spectra: deconvolution of Cu 2p XPS spectra of (b) Cu and (c) IL@Cu, deconvolution of C 1s XPS spectra of (d) Cu and (e) IL@Cu, and deconvolution of N 1s XPS spectra of (f) IL@Cu. (g) TGA thermograms of Cu, IL@Cu, and the IL.

The surface of the Cu powder was modified using the IL 1-ethyl-3-methyl imidazolium dicyanamide, and its functional group variations were characterized by using FTIR analysis. Figure 2a shows the FTIR spectra of pristine Cu, IL, and IL@Cu. The pristine Cu exhibits a characteristic absorption peak of the hydroxyl groups at 3440 cm^–1^. After surface modification, two peaks appeared at 3147 and 2982 cm^–1^, which are assigned to alkenyl C–H stretching vibrations and methylene and methyl C–H stretching vibrations, respectively; three peaks appeared at 2233, 1668, and 1313 cm^–1^, which are attributed to – C≡N, −N=C, and C–N– bonds, respectively. These results can be attributed to the IL grafted on the Cu surface after surface modification, thus exhibiting the structural characteristics of alkenyl, methylene, methyl, imidazole, and nitrile groups.^41,42^

The existence of the IL on the Cu surface is evident from the XPS results, as shown in Figure 2b–f. As presented in Figure 2b,c, the characteristic peaks of Cu_2p_ appeared at 931.9 and 951.8 eV, and the intensity of the Cu_2p_ peaks decreased after surface modification. The XPS spectrum of Cu shows two main peaks of Cu 2p_3/2_ and Cu 2p_1/2_ with one oxidation state of Cu^1+^. In contrast, the XPS spectrum of IL@Cu shows two main peaks of Cu 2p_3/2_ and Cu 2p_1/2_ with two oxidation states of Cu^1+^ and Cu^2+^.^43,44^ As shown in Figure 2d, the XPS spectrum of Cu shows the C_1s_ peak at 284.3 eV, which is assigned carbons (C–C). After surface modification, the intensity of the C 1s peak increased, and C≡N, C–N, and N=C–N peaks appeared in addition to the C–C peak,^45^ as shown in Figure 2e. Furthermore, a new peak was observed at 398.2 eV after surface modification, which is attributed to nitrogen [C≡N, N–(C)_3_, and C=N–C],^46,47^ as shown in Figure 2f.

The IL content on the Cu surface was determined by using TGA. Figure 2g shows the TGA thermograms of pristine Cu, the IL, and the IL@Cu. The weight losses of pristine Cu and IL@Cu at 800 °C calculated from the TGA thermograms were 0.56 and 7.76 wt %, respectively. These results indicate that the IL content on the Cu surface was 7.2 wt %.

**Figure 3:**
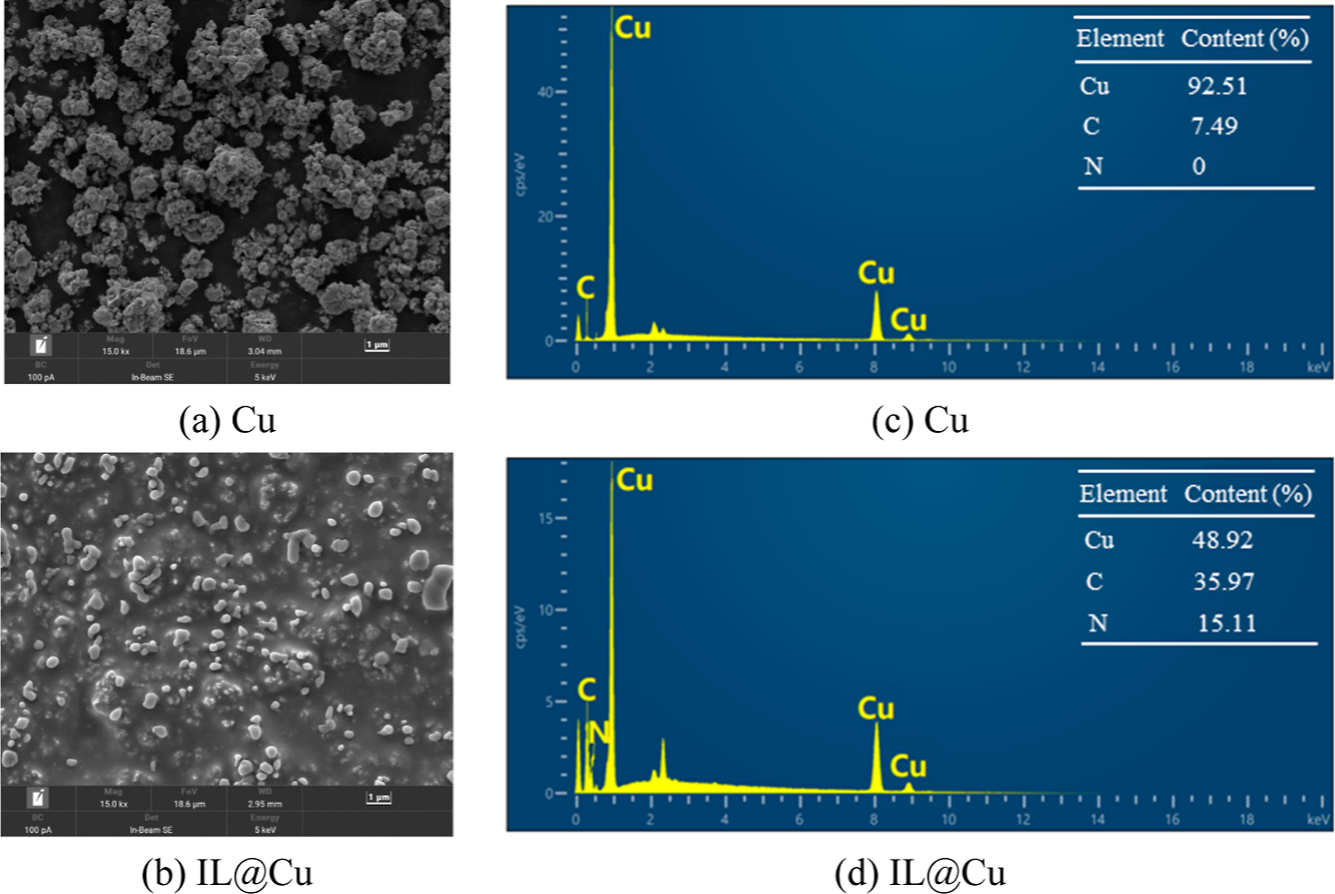
(a,b) SEM images of (a) Cu and (b) IL@Cu (magnification of 15,000×, scale bar of 1 μm). (c,d) EDX maps of (c) Cu and (d) IL@Cu.

To investigate the morphology of the Cu powder before and after surface modification and to assess the presence of the IL on the Cu surface, SEM–EDX observations were carried out. Figure 3a,b depicts the surface morphologies of pristine Cu and IL@Cu, respectively. As shown in Figure 3a, pristine Cu exhibits an aggregated surface. In contrast, IL@Cu presents a well-dispersed surface coated with a thin layer, as shown in Figure 3b.

Figure 3c,d shows the EDX maps of pristine Cu and IL@Cu, respectively. As presented in Figure 3c, the peaks at approximately 0.9, 8.1, and 8.9 keV are attributed to copper, while the peak at approximately 0.3 keV is attributed to carbon. A new peak associated with nitrogen at approximately 0.4 keV appeared after surface modification,^48^ as shown in Figure 3d. In detail, after surface modification, the copper content decreased from 92.51% to 48.92%, while the carbon and nitrogen contents increased from 7.49% and 0% to 35.97% and 15.11%, respectively. The above results confirm that the IL was successfully grafted onto the Cu surface.

### 3.2 Thermal Conductivity

**Figure 4:**
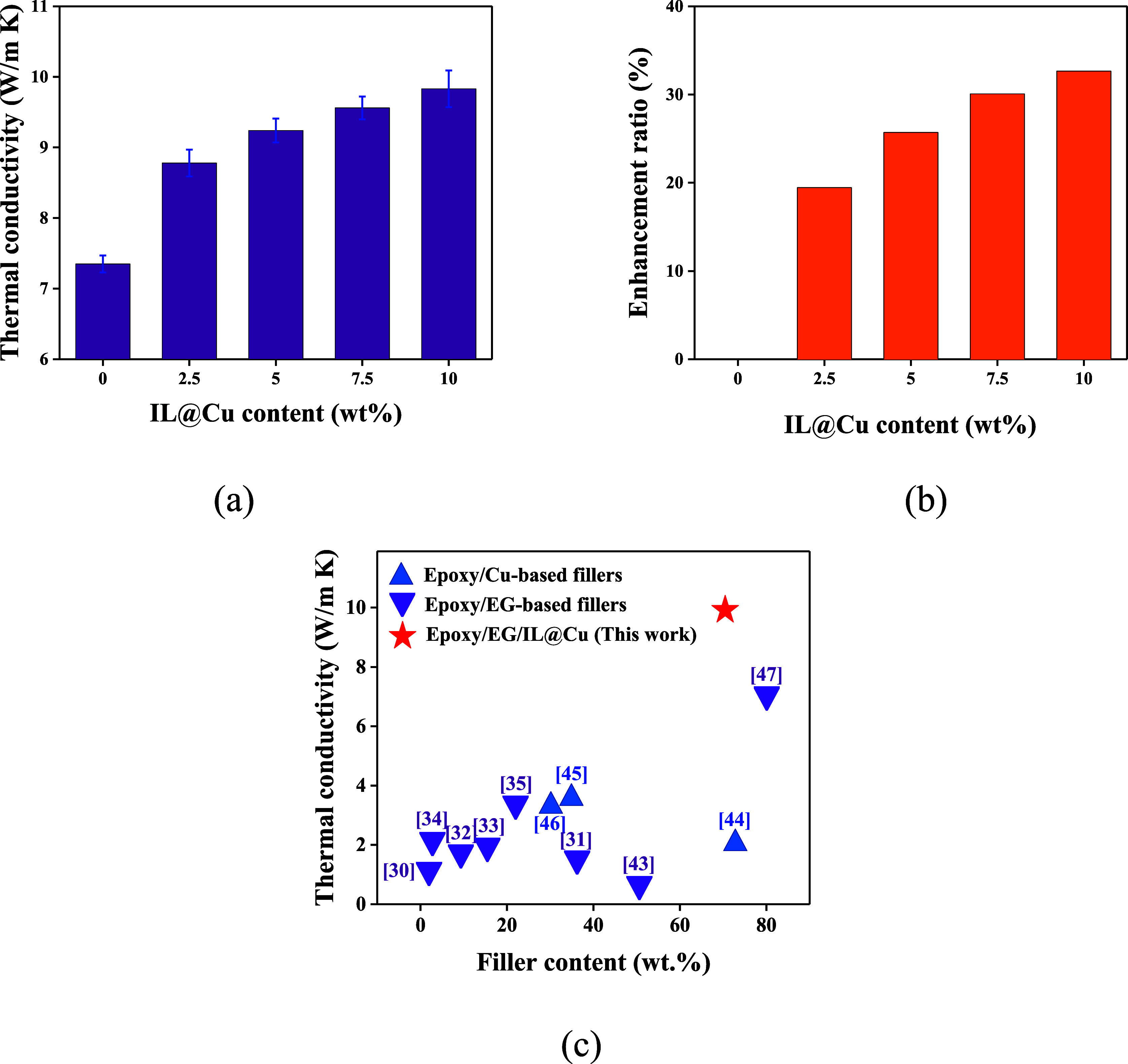
(a) Thermal conductivity and (b) thermal conductivity enhancement ratio of the DGEBA/EG/IL@Cu composites with various IL@Cu contents. (c) Comparison between the thermal conductivity of the as-prepared DGEBA/EG/IL@Cu composite and that of previously reported epoxy/Cu-based composites and epoxy/EG-based composites.

Figure 4a,b shows the variation in the thermal conductivity and thermal conductivity enhancement ratio, respectively, of the DGEBA/EG/IL@Cu composites as a function of the IL@Cu content. The thermal conductivity of the composites increased with an increasing IL@Cu content. Specifically, the DGEBA/EG composite containing 60 wt % EG exhibited a thermal conductivity of 7.35 W/(m·K). In contrast, the thermal conductivities of the DGEBA/EG/IL@Cu composites at 2.5 wt % IL@Cu and 10 wt % IL@Cu were 8.78 and 9.83 W/(m·K), respectively, which are 19.5% and 33.7% higher than that of the DGEBA/EG composite. These results can be explained as follows: EG has a lamellar structure, and a large number of pores exist between graphite sheets, which increases the interfacial thermal resistance. After surface modification of Cu using the IL, this IL is grafted onto the Cu surface and forms an organic film due to the electrophilicity of the IL cation, which improves the dispersion of Cu powder in the DGEBA matrix. Thus, Cu particles penetrate into pores of the graphite sheets to form a lamellar-sphere-lamellar structure and establish a thermal bridge between the graphite sheets, thereby reducing the interfacial thermal resistance and promoting the formation of continuous thermally conductive pathways. Moreover, the unique electron-rich conjugated structure of the IL on the Cu surface improves the thermal conductivity through an electronic mechanism.^49,50^ Therefore, the thermal conductivity of the DGEBA/EG/IL@Cu composite is improved by the addition of IL@Cu.

Figure 4c compares the thermal conductivity achieved in this work with that of previously reported epoxy/copper-based filler composites and epoxy/EG-based filler composites.^35−40,51−55^ As seen in this figure, other previously reported epoxy composites containing copper- and EG-based fillers possess relatively low thermal conductivity, reaching values of only 7 W/(m·K) even with high loadings of hybrid fillers. In contrast, our epoxy/EG/IL@Cu composites exhibit high thermal conductivity, reaching 9.83 W/(m·K).

### 3.3 Thermal Stability

**Figure 5:**
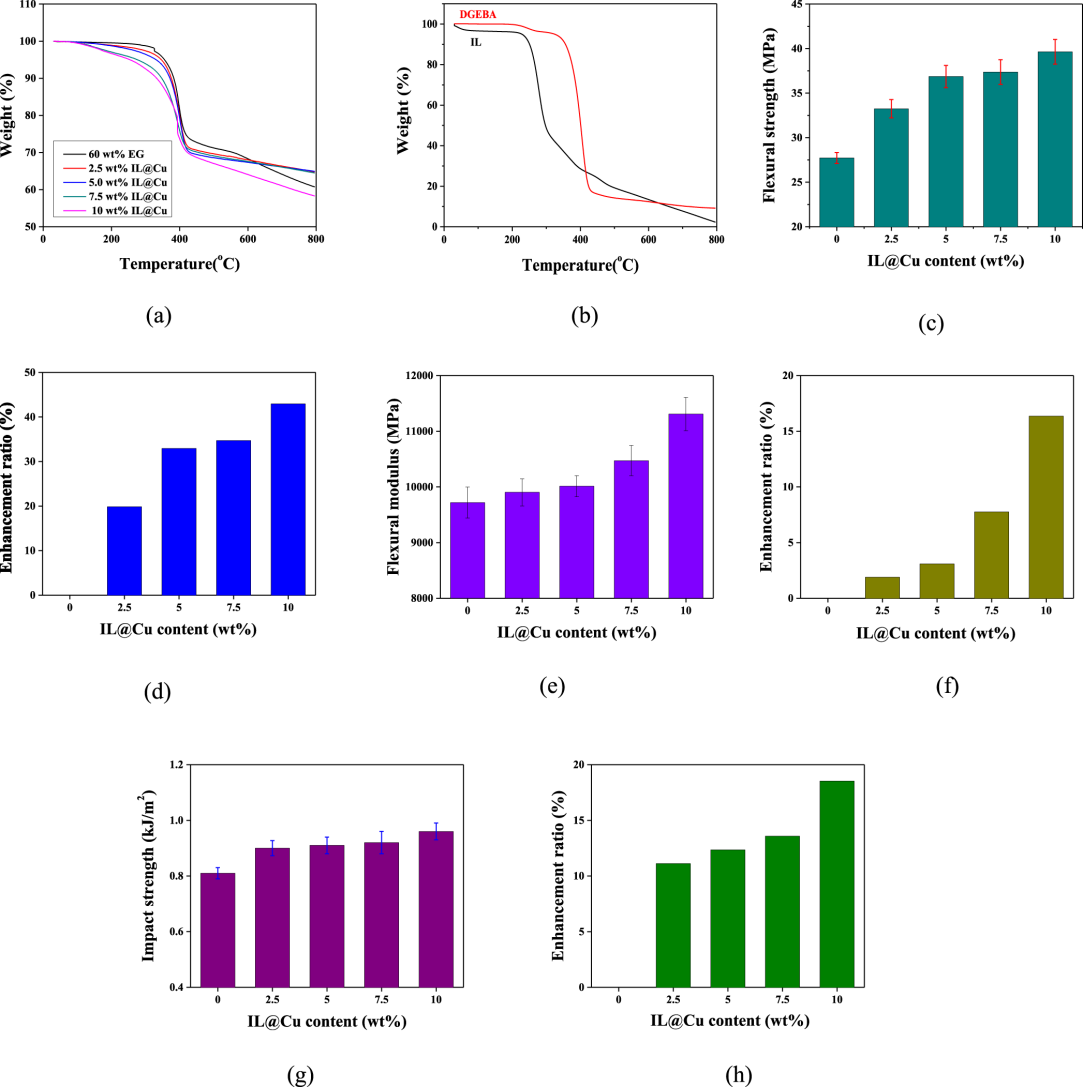
(a) TGA thermograms of the DGEBA/EG/IL@Cu composites as a function of the IL@Cu content. (b) TGA thermograms of DGEBA and the IL. (c) Flexural strength, (d) flexural strength enhancement ratio, (e) flexural modulus, (f) flexural modulus enhancement ratio, (g) impact strength, and (h) impact strength enhancement ratio of the DGEBA/EG/IL@Cu composites depending on the IL@Cu content.

The effect of the IL@Cu content on the thermal stability of the DGEBA/EG/IL@Cu composites was investigated by using TGA. Figure 5a shows the thermal degradation behavior of the composites as a function of the IL@Cu content. Thermal stability factors such as the 10% weight loss temperature (*T*_10%_) and the amount of char formation at 800 °C were calculated from the TGA thermograms,^56,57^ and the results are summarized in Table 1.

**Table 1:** Thermal Stability Factors of DGEBA/EG/Cu Composites as Obtained from TGA Thermograms

IL@Cu content (wt %)	*T*_10%_ (°C)	Char at 800 °C (%)
0	382.8	60.7
2.5	375.9	64.8
5	371.7	64.9
7.5	347.8	64.5
10	332.8	58.3

The *T*_10%_ values of the composites decreased with the addition of IL@Cu. That is, the *T*_10%_ value of the DGEBA/EG composite containing 60 wt % EG was 382.8 °C. In contrast, the *T*_10%_ values of the composites at 2.5 and 10 wt % IL@Cu were 375.9 and 332.8 °C, respectively, which were 6.9 and 50 °C lower than that of DGEBA/EG composite. This observation can be explained as follows: Cu particles have a good thermal conductivity. When the composite sample is heated, the temperature in the vicinity of the Cu particles is higher than that elsewhere in the sample, which accelerates the thermal degradation of the polymer around the Cu powder.^58,59^ Moreover, the IL grafted onto the Cu surface is an organic compound, which degrades more easily at a low temperature than polymers because it has a lower *T*_10%_ (250.9 °C) than that of pristine DGEBA (382.8 °C), as shown in the TGA thermograms of IL and DGEBA (Figure 5b). In addition, the amount of char formation of the composites at 800 °C is similar to that of the DGEBA/EG composite.

### 3.4 Flexural Properties

The flexural properties of the DGEBA/EG/IL@Cu composites were investigated by measuring their flexural strength and modulus. The effect of the IL@Cu content on the flexural strength and flexural strength enhancement ratio of the composites is presented in Figure 5c,d, respectively. The flexural strength of the composites increased significantly with the addition of IL@Cu. Specifically, the flexural strength of the DGEBA/EG composites was 27.9 MPa, whereas that of the composites at 10 wt % IL@Cu was 39.6 MPa, representing a 42% increase. This result can be explained as follows: upon external force, graphite sheets in the DGEBA/EG composites absorb energy by generating cracks until the deformation exceeds its limit. After surface modification, the Cu particles are dispersed uniformly in the DGEBA matrix and penetrate into the pores of the graphite sheet, resulting in a large number of microcracks in the DGEBA matrix, which can absorb more energy to resist deformation. Moreover, the nitrile and anionic amine groups of the IL on the Cu surface can bind with the epoxy resin, which improves the interfacial interactions between the Cu and DGEBA matrix.^60,61^ Therefore, the flexural strength of the DGEBA/EG/Cu composites increased with the addition of IL@Cu.

Figure 5e,f shows the variation in the flexural modulus and flexural modulus enhancement ratio, respectively, of the DGEBA/EG/IL@Cu composites with increasing IL@Cu content, indicating that the flexural modulus of the composites increased with the addition of IL@Cu. Specifically, the flexural modulus of DGEBA/EG composites was 9632 MPa, whereas that of the composites at 10 wt % IL@Cu was 11,309 MPa, representing a 17% increase. This result is attributed to the increased stiffness of the composites upon the addition of IL@Cu, resulting in an increased flexural modulus of the DGEBA/EG/IL@Cu composites.^62^

### 3.5 Impact Strength

The effect of the IL@Cu content on the impact strength and impact strength enhancement ratio of the DGEBA/EG/IL@Cu composites is presented in Figure 5g,h, respectively. The impact strength of the composites increased with increasing IL@Cu content. Specifically, the impact strength of DGEBA/EG composites was 0.81 kJ/m^2^, whereas that of the DGEBA/EG/IL@Cu composites at 10 wt % IL@Cu was 0.96 kJ/m^2^, representing a 19% increase. These results can be explained as follows: Cu particles penetrate into the pores of the graphite sheets and generate numerous microcracks in the DGEBA matrix that absorb energy upon external impact.^63,64^ Moreover, the nitrile and anionic amine groups of the IL on the Cu surface can be partially cross-linked with the DGEBA matrix, which improves the interfacial interactions between the Cu particles and DGEBA matrix, resulting in an increased impact strength of the DGEBA/EG/IL@Cu composites.

### 3.6 Morphology

**Figure 6:**
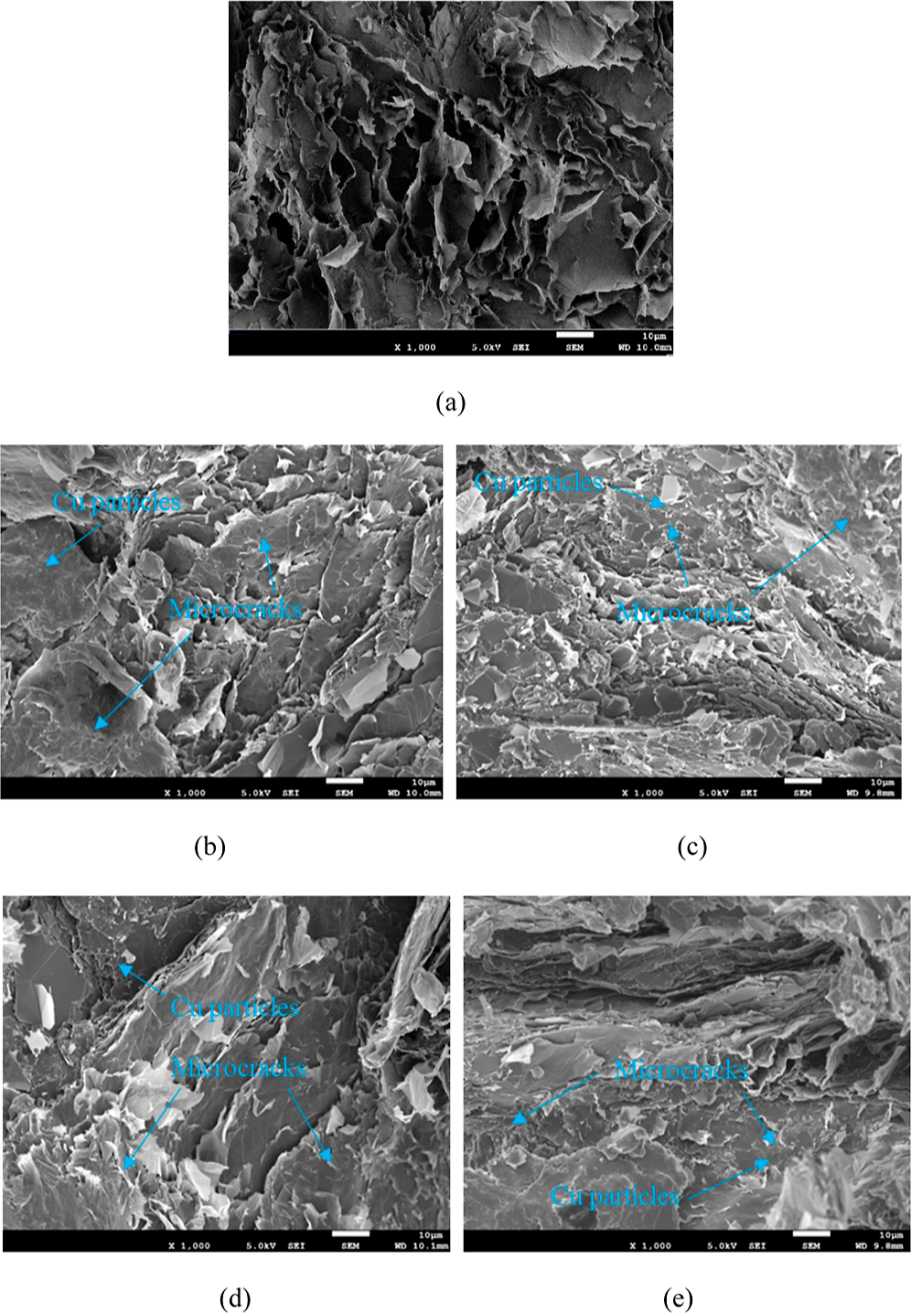
SEM micrographs of DGEBA/EG/IL@Cu composites: (a) 60 wt % EG; (b) 60 wt % EG + 2.5 wt % IL@Cu; (c) 60 wt % EG + 5 wt % IL@Cu; (d) 60 wt % EG + 7.5 wt % IL@Cu; and (e) 60 wt % EG + 10 wt % IL@Cu (magnification of 1000× , scale bar of 10 μm).

After the impact strength tests, the morphologies of the DGEBA/EG/IL@Cu composites with various IL@Cu contents were investigated by SEM; the corresponding SEM images of the fractured surfaces are depicted in Figure 6. As shown in Figure 6a, due to their low impact strength, the DGEBA/EG composites appear as sheet-shaped blocks that peel away from the fracture surface under an external force.^65^ In contrast, with the addition of IL@Cu to the DGEBA/EG composites, the sheet-shaped blocks become less abundant, and numerous microcracks are generated; the generated microcracks absorb more external energy when an external force is applied, which increases the impact strength of the DGEBA/EG/IL@Cu composites,^66^ as shown in Figure 6b–e. In addition, the fracture surface of the DGEBA/EG/IL@Cu composites retains sheet-shaped blocks similar to those of the DGEBA/EG composites, and the Cu particles and graphite sheets together form a continuous thermally conductive pathway in the DGEBA matrix, thereby improving the thermal conductivity of the resulting DGEBA/EG/IL@Cu composites.^52,67^

## 4 Conclusions

In this study, DGEBA/EG/IL@Cu composites with high thermal conductivity and mechanical properties were prepared via hot blending and compression-curing processes. The thermal conductivity, thermal stability, flexural properties, impact strength, and morphologies of the composites were investigated. The Cu surface was modified using an IL, and its surface characteristics were characterized by using FTIR, XPS, TGA, and SEM–EDX measurements. The results indicated that the thermal conductivity of the DGEBA/EG/IL@Cu composites increased from 7.35 to 9.86 W/m·K (34% increase) with increasing IL@Cu content from 0 to 10 wt %. The thermal stability of the composites decreased with the addition of IL@Cu. The flexural strength and flexural modulus of the composites at 10 wt % IL@Cu were 39.6 and 11,309 MPa, respectively, which are 42% and 18% higher than those of the DGEBA/EG composites. The impact strength of the composites increased from 0.81 to 0.96 kJ/m^2^ (19% increase) with the addition of 10 wt % IL@Cu. The SEM results of the DGEBA/EG/IL@Cu composites revealed sheet-shaped blocks with numerous microcracks on the fracture surfaces, which form a continuous thermally conductive pathway within the DGEBA matrix.
